# Differentiation Potential and Tumorigenic Risk of Rat Bone Marrow Stem Cells Are Affected By Long-Term In Vitro Expansion

**DOI:** 10.4274/tjh.galenos.2019.2019.0100

**Published:** 2019-11-18

**Authors:** Erdal Karaöz, Filiz Tepeköy

**Affiliations:** 1 İstinye University Faculty of Medicine, Department of Histology and Embryology, İstanbul, Turkey; 2 İstinye University Center for Stem Cell and Tissue Engineering Research and Practice, İstanbul, Turkey; 3 Center for Regenerative Medicine and Stem Cell Research and Manufacturing (LivMedCell), İstanbul, Turkey; 4 Altınbaş University Faculty of Medicine, Department of Histology and Embryology, İstanbul, Turkey

**Keywords:** Bone marrow, Differentiation, Long-term culture, Mesenchymal stromal cells, Stemness, Telomerase

## Abstract

**Objective:**

Mesenchymal stem cells (MSCs) have the capacity for extensive expansion and adipogenic, osteogenic, chondrogenic, myogenic, and neural differentiation in vitro. The aim of our study was to determine stemness, differentiation potential, telomerase activity, and ultrastructural characteristics of long-term cultured rat bone marrow (rBM)-MSCs.

**Materials and Methods:**

rBM-MSCs from passages 3, 50, and 100 (P3, P50, and P100) were evaluated through immunocytochemistry, reverse transcription-polymerase chain reaction, telomerase activity assays, and electron microscopy.

**Results:**

A dramatic reduction in the levels of myogenic markers actin and myogenin was detected in P100. Osteogenic markers Coll1, osteonectin (Sparc), and osteocalcin as well as neural marker c-Fos and chondrogenic marker Coll2 were significantly reduced in P100 compared to P3 and P50. Osteogenic marker bone morphogenic protein-2 (BMP2) and adipogenic marker peroxisome proliferator-activated receptor gamma (Pparγ) expression was reduced in late passages. The expression of stemness factor Rex-1 was lower in P100, whereas Oct4 expression was decreased in P50 compared to P3 and P100. Increased telomerase activity was observed in long-term cultured cells, signifying tumorigenic risk. Electron microscopic evaluations revealed ultrastructural changes such as smaller number of organelles and increased amount of autophagic vacuoles in the cytoplasm in long-term cultured rBM-MSCs.

**Conclusion:**

This study suggests that long-term culture of rBM-MSCs leads to changes in differentiation potential and increased tumorigenic risk.

## Abstract (tr)

**Amaç:**

Mezenkimal kök hücreler (MKH) in vitro uzun süreli ekspansiyon, adipojenik, osteojenik, kondrojenik, miyojenik ve nöral farklılaşma potansiyeline sahiptir. Çalışmamızın amacı uzun süre kültüre edilen sıçan kemik iliği (sKİ)-MKH’lerinin kök hücre niteliklerini, farklılaşma potansiyellerini, telomeraz aktivitelerini ve ultrayapısal özelliklerini belirlemektir.

**Gereç ve Yöntemler:**

3., 50. ve 100. pasajlardan (P3, P50 ve P100) elde edilen sKİ-MKH’leri, immünohistokimya, revers-transkriptaz polimeraz zincir reaksiyonu, telomeraz aktivite analizleri ve elektron mikroskopi ile değerlendirilmiştir.

**Bulgular:**

P100’de miyojenik belirteçlerden aktin ve miyogenin seviyelerinde düşüş gözlemlenmiştir. Osteojenik belirteçler Coll1, osteonektin (Sparc) ve osteokalsin ile nöral belirteç c-Fos ve kondrojenik belirteç Coll2 P100’de P3 ve P50’ye kıyasla önemli ölçüde azalmıştır. Osteojenik belirteç kemik morfojenik protein-2 (BMP2) ve adipojenik belirteç peroksizom proliferatör ile aktive olan reseptör gamma (Pparγ) geç pasajlarda düşüş göstermiştir. Kök hücre belirteçlerinden Rex-1’in ekspresyonu P100’de düşüş gösterirken, Oct4 ekspresyonunun P50’de P3 ve P100’e göre düşüş gösterdiği belirlenmiştir. Uzun süre kültüre edilen hücrelerdeki artmış telomeraz aktivitesi tumorijenik riske işaret etmektedir. Elektron mikroskopik değerlendirmeler, uzun süre kültüre edilen sKİ-MKH sitoplazmasında düşük sayıda organel ve artmış oranda otofajik vaküol gibi utrayapısal değişiklikler ortaya koymuştur.

**Sonuç:**

Bu çalışma, sKİ-MKH’lerinin uzun süreli kültüre edilmesinin bu hücrelerin farklılaşma potansiyelinde değişikliklere ve tümörijenik riskin artmasına neden olduğunu göstermiştir.

## Introduction

Mesenchymal stem cells (MSCs) have the capacity for extensive expansion in vitro and are able to undergo adipogenic, osteogenic, chondrogenic, myogenic, and neural differentiation [1,2,3]. MSCs can be obtained from several sources, such as placental tissue [4], amniotic fluid [5], cord blood [6,7], adipose tissue [8,9], and dental pulp [10]. However, bone marrow aspiration remains the source of choice for MSCs in most laboratories [11,12]. The secretion of a broad range of bioactive molecules is believed to be the main mechanism by which MSCs achieve their therapeutic effects and these can be divided into eight main categories: immunomodulation, anti-apoptosis, angiogenesis, support of the growth and differentiation of local stem and progenitor cells, anti-scarring, chemoattraction, gene transfer, and exosomes [13,14,15,16,17,18,19].

A sufficient quantity of stem cells can be obtained by in vitro expansion in order to be used in clinical applications [20]. However, during long-term cultures of stem cells, several abnormalities were recorded, such as increased telomerase activity and changes in the expression of genes regarding cell regulation, apoptosis, and senescence due to increased cell doublings and culture times [21,22,23,24]. Thus, we proposed that long-term expansion of MSCs in vitro might be associated with tumorigenic risk. MSCs were reported to promote tumor progression and metastasis in a number of studies [25,26,27,28], while other studies suggested that MSCs suppress tumor growth [29,30,31]. Spontaneous transformation was not observed during in vitro expansion of human MSCs (hMSCs) [32,33,34,35]. However, there are reports providing evidence that murine bone marrow (BM)-MSCs [36] as well as adipose-derived hMSCs [37] displayed malignant transformation in vitro. It was suggested that the tendency of hMSCs to undergo malignant transformation was caused by the genomic plasticity of undifferentiated hMSCs allowing their longevity [38]. BM-MSCs were also reported to be associated with the in vivo growth of colon cancer, lymphoma, and melanoma cells [26,39,40]. MSCs were found to transform into tumor-associated fibroblasts, constructing a fibrovascular network for the tumors [41]. On the other hand, BM-MSCs were also shown to suppress tumorigenic cells in vivo [30,42].

The aim of the current study was to evaluate long-term (18 months, 100 passages) cultured rat bone marrow (rBM)-MSCs in terms of stemness and differentiation characteristics as well as cell cycle progression and telomerase activity in order to determine their lineage differentiation potential and tumorigenic risk under in vitro conditions.

## Materials and Methods

### Isolation and Culture of rBM-MSCs

The animals (8-week-old male Wistar rats) were anesthetized with Ketalar (Pfizer) and killed by cervical dislocation for rBM-MSC isolation. Animal housing and experiments were approved by the local animal care committee (Kocaeli University, HAEK/33-4) according to the institutional guidelines and national animal welfare standards. rBM-MSCs were obtained from both femurs and tibias of the rats as described in our previous study [43].

For each passage the cells were plated similarly and grown to confluency of 70%. Passages were performed until 100 passages and the below-mentioned analyses were performed for passages 3, 50, and 100.

### Immunocytochemistry and Immunofluorescence Staining

The streptavidin-peroxidase method (UltraVision Plus Large Volume Detection System Anti-Polyvalent, HRP Immunostaining Kit, Thermo Scientific, UK) was used for immunocytochemistry analysis as described previously [10]. Immunofluorescence staining was applied as indicated in our previous study [10]. The primary antibodies listed in Table 1 were used for immunocytochemistry and immunofluorescence stainings.

### Reverse Transcription-Polymerase Chain Reaction (RT-PCR)

Total RNA was isolated from rBM-MSCs (passages 3, 100, and 150) according to the manufacturer’s instructions (QIAGEN, USA). RT-PCR analysis was performed as described in our previous study [44] and bands were quantified using NIH image analysis software (ImageJ Version 1.36b, National Institutes of Health, Bethesda, MD, USA) as described previously [45].

### Telomerase Activity

Telomerase activity was detected by applying a conventional telomeric repeat amplification protocol (TRAP) using the TRAP TeloTAGGG PCR enzyme-linked immunosorbent assay kit (Roche, Mannheim, Germany). The TRAP method was applied as described previously [46].

### Electron Microscopy

rBM-MSCs at passages 3, 50, and 100 were prepared for electron microscopic analysis. The samples were fixed and embedded as described previously [47]. Ultrathin sections were observed with a transmission electron microscope (Carl Zeiss Libra 120).

### Statistical Analysis

The data obtained from ImageJ for RT-PCR bands were analyzed with non-parametric ANOVA on ranks (Kruskal-Wallis test) and parametric one-way ANOVA (Holm-Sidak method). The values are presented as mean ± SEM. Statistical calculations were performed using Sigma Stat for Windows, version 3.0 (Jandel Scientific Corp., San Rafael, CA, USA). Statistical significance was defined as p<0.05.

## Results

### Immunolocalization of Differentiation Markers in Long-Term Cultured rBM-MSCs

**Figure 1:**
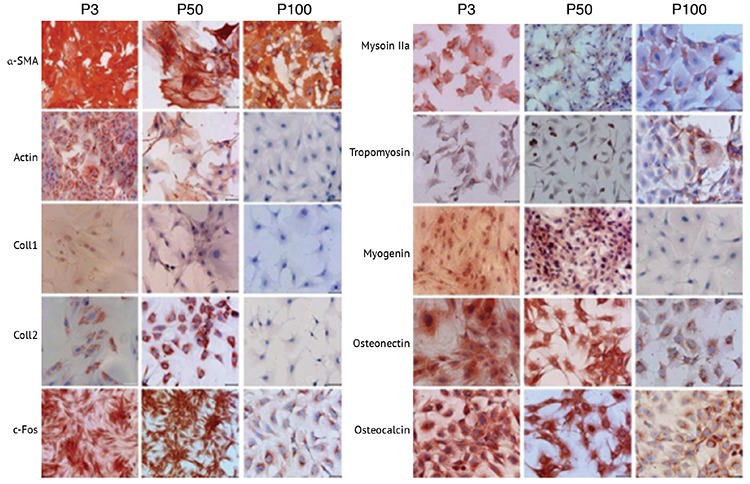
Lineage differentiation marker localizations in cultured rat bone marrow mesenchymal stem cells. P3: Passage 3. P50: Passage 50. P100: Passage 100. Nuclei were counterstained with hematoxylin. All experiments were repeated 3 times. Scale bars: 50 μm.

Immunocytochemistry analysis in the current study showed that levels of particular myogenic markers including a-SMA and tropomyosin remained similar both in early and late passages. There was a dramatic reduction in actin, myosin IIa, and myogenin levels in passage 100 when compared to passages 3 and 50. Osteogenic markers including Coll1, osteonectin, and osteocalcin as well as neural marker c-Fos and chondrogenic marker Coll2 were reduced in passage 100 compared to passages 3 and 50 (Figure 1; Table 2).

**Figure 2:**
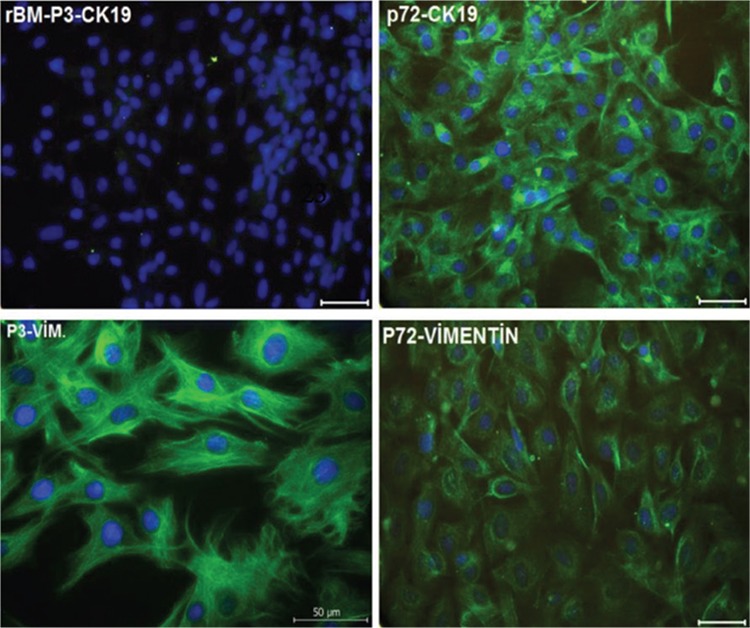
Localizations of cytokeratin 19 (green) and vimentin (green) in cultured rat bone marrow mesenchymal stem cells. P3: Passage 3. P72: Passage 72. Nuclei were labeled with DAPI (blue). All experiments were repeated 3 times. Scale bars: 50 μm.

Immunofluorescence analysis revealed that expression of epithelial marker CK-19 was increased after passage 70, while expression of mesenchymal marker vimentin was decreased after passage 70 when compared to passage 3 (Figure 2).

### Gene Expression Profiles of Long-Term Cultured rBM-MSCs

The expressions of the stemness factors as well as adipogenic, chondrogenic, osteogenic, myogenic, and neural differentiation markers were detected in long-term cultured rBM-MSCs by RT-PCR analysis using specific primer sets (Table 3).

The expressions of stemness factors Rex-1 and Oct4 were identified in rBM-MSCs in all passages (P3, P50, and P100). Rex-1 expression level was increased in P50 and was decreased in P100 to a lower level than in P3. Oct4 was decreased in P50 compared to P3. Although it was found to be increased in P100, its expression in P100 was lower than in P3. Chondrogenic marker Sox9 was expressed in both early and late passages, and its expression was increased in P50 and significantly decreased in P100. The expressions of differentiation markers of precursor osteoblasts such as osteopontin (Opn/Ssp1), run-related transcription factor 2 (Runx2), and osteonectin (Sparc) were increased in P50 and were significantly reduced in P100. BMP2 was detected to be expressed at significantly lower levels both in P50 and P100 compared to P3, whereas the BMP4 level was lower only in P50.

**Figure 3:**
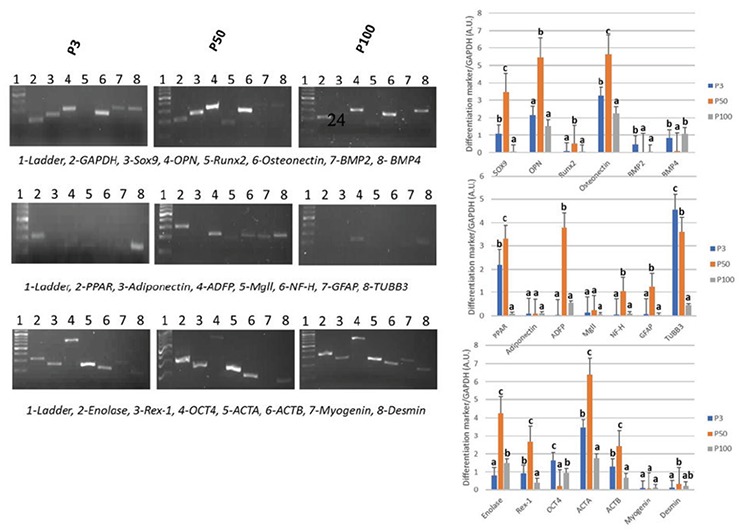
Reverse transcription-polymerase chain reaction bands and graphics of mathematical values of ImageJ evaluations of embryonic stem cell (Rex-1 and Oct4) and differentiation (Sox-9, osteopontin, Runx2, osteonectin [SPARC], BMP-2, BMP-4, PPAR, adiponectin, ADFP, MgII, NF-H, GFAP, TUBB3, Eno2, ACTA, ACTB, myogenin, and desmin) markers in cultured rat bone marrow mesenchymal stem cells. Values are presented as mean ± SEM. Different letters mark statistical significance (p<0.05) (one-way ANOVA, Holm-Sidak method). P3: Passage 3. P50: Passage 50. P100: Passage 100. All experiments were repeated 3 times.

Expression of the adipogenic marker Pparγ was decreased in P100 compared to P3 and P50. Adiponectin and monoglyceride lipase (MgLL) expressions were detected to be similar in all passages. ADFP was expressed in all three passages, with a higher level in P50. Neurofilament heavy chain (NF-H) and glial fibrillary acidic protein (GFAP) expressions were higher in P50 compared to P3 while they were decreased in P100. Neuroprogenitor cell marker β3-tubulin (TUBB3) was significantly decreased in P100 compared to P3 and P50. Another neuroprogenitor cell marker, gamma enolase (Eno2), was increased in P50 and reduced in P100 compared to P50. Precursor myoblast markers α-smooth muscle actin (Acta2) and ActB were increased in P50 and decreased in P100, whereas desmin (Des) and myogenin (Myog) expression levels were similar in all passages (Figure 3).

### Telomerase Activity

**Figure 4:**
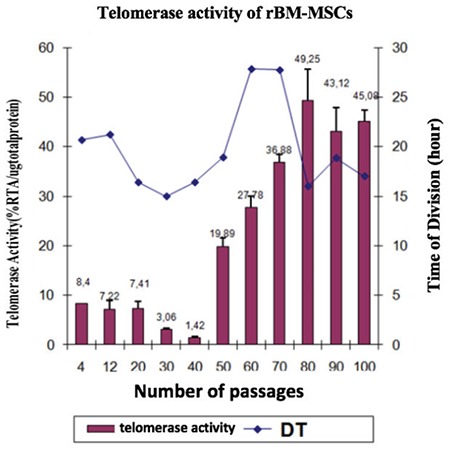
Telomerase activity assessment of cultured rat bone marrow mesenchymal stem cells. Values are presented as mean ± SEM. All experiments were repeated 3 times.

Relative telomerase activities (RTAs) of rBM-MSCs (P3, P50, and P150) were measured and the calculations were normalized to 1 µg of total protein equivalent. The results for rBM-MSCs at passages 3, 50, and 100 were found as 8.4, 19.89, and 45.09 RTA/µg total protein, respectively. According to these data, rBM-MSCs at later passages show a higher rate of telomerase activity (Figure 4).

### Ultrastructural Characteristics

**Figure 5:**
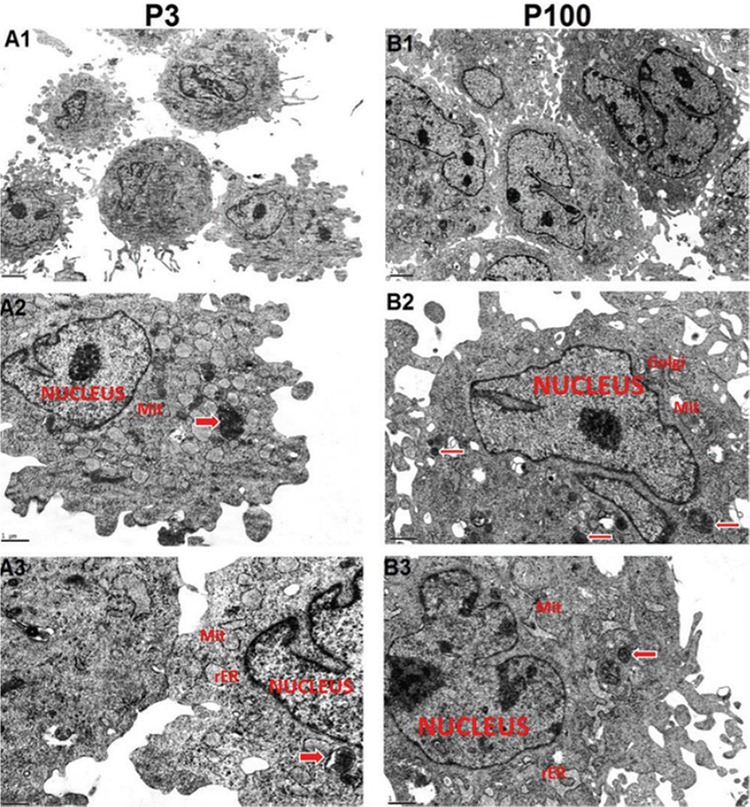
Electron micrographs of cultured rat bone marrow mesenchymal stem cells. P3: Passage 3. P100: Passage 100. Rough endoplasmic reticulum (rER), mitochondria (Mit), Golgi apparatus (Golgi), and autophagic vacuoles (arrows) are marked. All experiments were repeated 3 times.

rBM-MSCs from both early and late passages showed pale, eccentric, irregularly shaped, and large euchromatic nuclei with one or more nucleoli located near the perinuclear cisternae. The cell cytoplasms from passage 3 had an intensely stained inner zone rich in elongated mitochondria and rough endoplasmic reticulum (rER) cisternae and a relatively peripheral zone poor in organelles. The rER cisternae were dilated and contained moderately electron-dense material. Aggregates of a few lipid droplets, granules, and glycogen were also observed. Numerous thin pseudopodia were observed on the cell surfaces. rBM-MSCs from late passages contained a smaller number of organelles and increased amount of pseudopodia on the cell surfaces. Empty vacuoles in the cytoplasm were observed to be increased in rBM-MSCs from late passages with respect to early passages. Free ribosomes were observed in the cytoplasms of cells from both early and late passages. These results constitute the first comparative and comprehensive detailed report of ultrastructural characteristics on long term cultured rBM-MSCs (Figure 5).

## Discussion

There are conflicting results in the literature regarding malignant transformation of MSCs during in vitro culture. A number of reports proved the transformation of these cells [38,48,49,50], whereas certain studies found a relation with aneuploidy [51,52,53,54] and genetic mutations [55] while other studies suggested that these cells do not undergo transformation after long-term expansion [34]. In the current study we performed long-term, non-stop culture of rBM-MSCs for 18 months including 100 passages. These long-term cultured cells were examined for stemness factors as well as myogenic, chondrogenic, adipogenic, osteogenic, and neurogenic differentiation markers; epithelial and mesenchymal cell markers; telomerase activity; and ultrastructural characteristics. Interestingly, these cells showed higher expressions of CK-19 and lower expressions of vimentin after passage 70, signifying mesenchymal-to-epithelial transition in late passages. Previous studies regarding long-term culture of hMSCs also identified transformation of spindle-shaped cells into round epithelial-like cells that had an increased nucleus-to-cytoplasm ratio [38]. In previous studies it was shown that long-term cultures of both human [56] and rabbit [57] MSCs resulted in cellular senescence. Long-term expanded senescent cells were shown to have reduced differentiation potential, which led to restriction in MSC expansion for therapeutic applications [56,58,59]. Though we have found that rBM-MSCs preserve stemness factors even in late passages, they lack particular differentiation markers after long-term culture, highlighting their limited differentiation potential.

As an indication of the reduced adipogenic differentiation capacity of rBM-MSCs during long-term culture, in the current study we detected that expression of adipogenic marker PPAR-c was significantly decreased in late passages. PPAR-c is known to induce adipogenesis [60]. PPAR-c suppression was detected to cause generation of osteoblasts rather than adipocytes from BM progenitors [61]. After long-term in vitro expansion, although BM-MSCs were unable to display adipogenic differentiation, they were shown to have osteogenic differentiation potential [58].

Furthermore, it was also shown that osteogenic differentiation potential does not depend on the age of the donor [62]. In our study, expression levels of most of the osteogenic markers, including BMP-2, were significantly reduced during long-term culture. Coll1, osteonectin, osteocalcin, and Runx2 were detected to be reduced, especially in P100. Additionally, a dramatic reduction in chondrogenic marker SOX9 levels was detected, as well as a decrease in Coll2 levels in late passages. Thus, our results including in vitro expanded stem cells showed that the osteogenic and chondrogenic potential of long-term cultured stem cells might be disrupted. In previous studies, it was reported that human adipose-derived stem cells were able to differentiate into osteogenic cells, but this ability was reduced after long-term in vitro expansion [63].

The level of neural marker TUBB3 was gradually reduced in late passages and was detected to be significantly lower in P100. The c-Fos level was also decreased in P100. Although NF-H and GFAP levels were increased in P50, they were detected to be reduced in P100. Thus, the data obtained in this study indicate that the neurogenic differentiation potential of rBM-MSCs might be affected by long-term culture. Particular myogenic differentiation markers including myogenin and desmin were detected to be expressed in low levels both in early and late passages, whereas myogenin was found to be reduced during the late passages. Levels of a-SMA and tropomyosin were also detected to be similar in both early and late passages. However, myogenic markers ACTa, ACTb, and myosin IIa were detected to be reduced in P100. These results indicate that, in order to evaluate the lineage differentiation of stem cells, particular markers should be assessed in terms of both gene and protein levels.

Autophagy has been shown to effect the inhibition of continuous growth of precancerous cells and suppression of cancer [64]. As reviewed by Kocaturk et al. [65], autophagy leads to the removal of damaged macromolecules or organelles, such as mitochondria [66], ER [67], ribosomes [68], and lipid droplets [69]. We have also revealed ultrastructural changes in BM-MSCs at late passages, including a smaller number of organelles as well as a high number of autophagic vacuoles in the cytoplasm, which might be an indication of tumorigenic cells with increased rates of autophagy. Telomere length displays the proliferative potential of somatic cells [70]. Telomerase activity levels and telomere lengths were investigated in order to examine the safety of long-term cultured hMSCs in previous studies [56,71]. There are conflicting reports regarding the telomerase activity of these cells. It was shown that telomerase activity in hMSCs during long-term culture was not altered and remained at a very low level, and telomere lengths of hMSCs were remarkably decreased at late passages [71], while other studies showed that the telomerase activity of cultured hMSCs decreased and these cells displayed telomere shortening during serial passaging [56,72,73]. Some reports revealed senescence in the culture ultimately [55]. However, in our study, we have found increased telomerase activity of BM-MSCs in late passages, consistent with particular reports revealing several abnormalities in long-term cultured MSCs including increased telomerase activity [21,22].

Rodent BM-MSCs and hMSCs have displayed some common surface antigens such as CD29, CD90, and CD105, used for MSC characterization [74]. Gene expression profiling of MSCs from rodents has revealed a high degree of concordance with hMSCs [75]. The changes in gene expressions and protein levels of rBM-MSCs during long-term culture might also be possible for hMSCs and we believe that for clinical applications following long-term culture of these cells, data obtained from both humans and rodents must be considered.

## Conclusion

The data obtained from this study reveal that long-term culture of rBM-MSCs leads to changes in the MSC characteristics of these cells as well as increased tumorigenic risk via increased telomerase activity. In order to provide efficiency of differentiation potential and safety regarding tumor formation risk of cultured MSCs for cellular therapy, further phenotypic and functional investigations as well as genetic characterizations of MSCs must be conducted.

## Figures and Tables

**Table 1:**
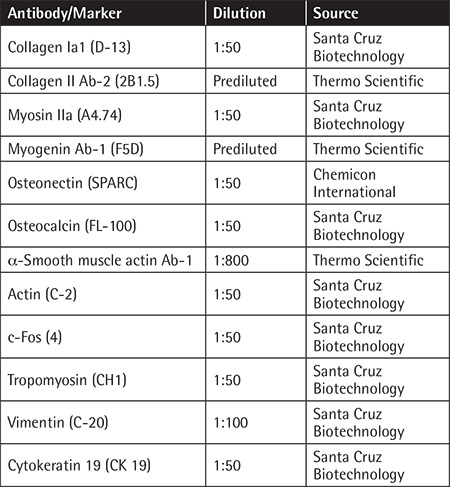
Primary antibodies used for immunocytochemistry and immunofluorescence analysis. Antibody/Marker Dilution

**Table 2:**
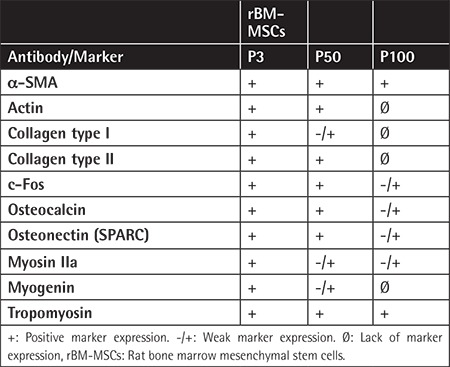
Immunocytochemical properties of rBM-MSCs.

**Table 3:**
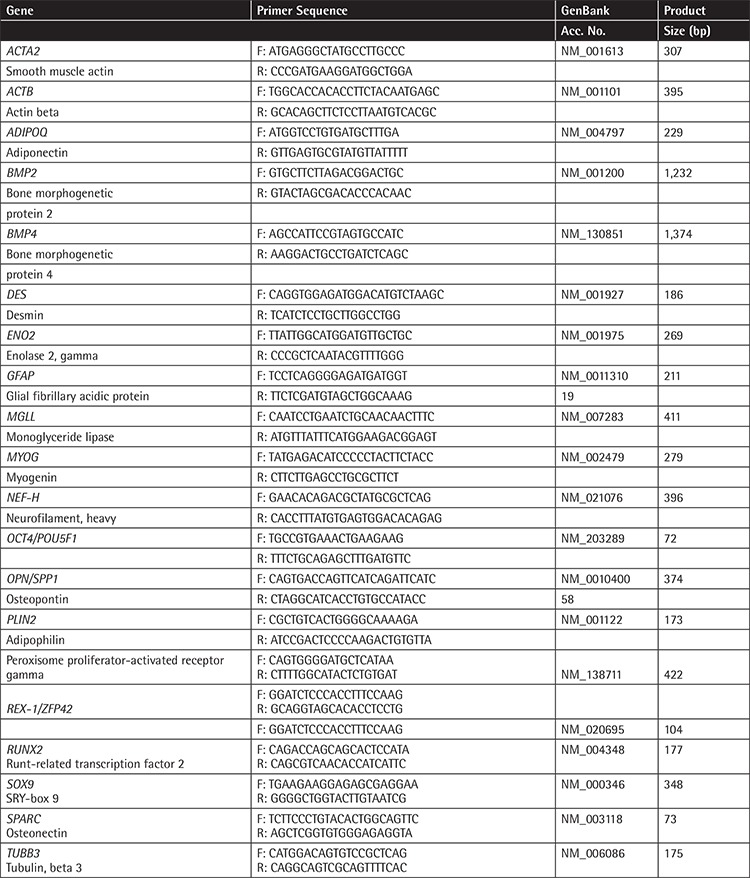
Primers used in polymerase chain reaction analysis.
